# The Behavior of Tendon Reflexes in Health and Disease

**Published:** 1892-10

**Authors:** William C. Krauss

**Affiliations:** B. S., Dr. Med. (Berlin), of Buffalo, N. Y., Member of the American Neurological Association; 382 Virginia Street


Buffalo Medical and Surgical Journal

Vol. XXXII. OCTOBER, 1892. No. 3.

### Original Communications.

### THE BEHAVIOR OF TENDON REFLEXES IN HEALTH AND DISEASE.

By WILLIAM C. KRAUSS. B. S., Dr. Med. (Berlin), of Buffalo, N. Y.,
Member of the American Neurological Association.

If an isolated muscle in the animal body receive an impulse from the higher nerve centers, it will shorten, and cause some change of position of its movable insertion. This is termed muscular contraction, and is influenced by the will or desire of the individual, and hence we speak of it as a voluntary act. The muscle thus subjected is a voluntary muscle, and the work accomplished is the function of that muscle. Now, if that muscle be removed from its bony attachments, stretched between two supports, one movable and one fixed, and subjected to the action of an irritant, it will likewise contract, and its movable insertion will change position as in the living body. This is termed muscular irritability, and is independent of the will or volition of the individual, hence involuntary. This irritability, sometimes designated as myotatic, is the foundation or starting-point of tendon, or, better, muscular reflexes. The same involuntary contraction will be produced if the nerve be subjected to the same influences as was the muscle. The irritant or stimulus may be mechanical, chemical, thermal, or electrical. Tersely stated, a reflex is the contraction of a muscle independent of the will, produced by the action of a stimulus upon its fibers or its nerve fibers. The mechanism of the reflexes which we are about to study, although categorically the same, is somewhat more complex and complicated.

*Historical.*—The study of the reflexes is an old one, and has attracted the attention of medical minds for centuries. The reflex action of the pupils to light was known to Galen. Descartes was acquainted with the reflex phenomena, but could not account for their production. In fact, in early times the whole subject was classed as pertaining to the sympathetic system.

Haller, the father of German physiology, found that irritation of some tissues caused sensation, and that of others caused movement or contraction.

Swamnerdam (1637–1680) noticed the reflex movements of sleeping animals and men when the skin was gently tickled.

Willis, a student of Descartes, likened the reflex process to that of reflected sound in echo, but differed from Descartes in believing the periphery, as well as the brain, the seat of the reflex process.

Whytt (1714–1766) seemed to have a clearer conception of this phenomenon, for he wrote as follows: A certain power or influence lodged in the brain, spinal marrow, and nerves, is either the immediate cause of contraction of the muscles of animals, or, at least, necessary to it.

Unger (1727–1799) pointed out more clearly than had been done before, the path of sensory impulses from the periphery to the brain.

Prochaska's (1749–1820) idea of reflex action was as follows: External impressions made on sensitive nerves are propagated with great velocity to their origin, where, when they have arrived, are reflected—and pass into certain and corresponding motor-nerves, which, propagated to muscles, excite certain and determinate movements.

Bell, in demonstrating that the posterior roots of the spinal cord are sensory and the anterior motor, made the most important discovery in reflex action that had been made up to his time (1811). In 1809, Walker, a Scotch anatomist, proved the converse of Bell's law, viz., that anterior roots are sensory and the posterior motor.

Magendie (1783–1855) made the same discovery Bell did, and claimed priority. This claim is still held by many of the French scientists. Müller (1834) established Bell's law in Germany, and between Bell, Magendie, and Müller, the anatomical elements concerned in reflex action were made known, viz., a centripetal and a centrifugal nerve, with their portion of the spinal cord.

The first to elaborate a mechanical theory of reflex action was Marshall Hall (1790–1857). Besides the central system and the ganglionic, he assumed a third, the true spinal system, or an excito-motor system.

Pflüger, in 1855, formulated his well-known laws of reflex action.

Lotze carried the theory of Marshall Hall still higher, and perfected it.

The clinical importance of tendon reflex action was unknown, however, until the latter part of the present century. In 1858, Brown-Séquard first described ankle-clonus as occurring in the dog and guinea pig. The importance and weight which this observer lent the subject induced Vulpian and Charcot to make observations on their patients at the Salpêtrière, Paris (1866). They called particular attention to the diagnostic importance of ankle-clonus in diseases of the spinal cord. Since then, numerous contributions have emanated from the same source, and the Salpêtrière has become the center of modern neurological research. The German school was not long, however, in grasping the significance of this work. Westphal, in his studies on locomotor ataxia at the Charitè, Berlin, first discovered the absence of the patellar reflex in this disease (1874). Westphal's symptom, so-called, has passed into history. Contemporaneously with Westphal, but yet independent, appeared Erb, of Heidelberg, who began the study of the mechanism of tendon reflex, and its behavior in the various affections of the brain and cord. An army of workers, scattered throughout the civilized world, has been engaged since then trying to unravel the true nature and full significance of this strange phenomenon.

The scientific study of this phenomenon dates from the discoveries of Westphal and Erb in 1874, and the tendon reflex is now recognized as an important diagnostic factor in the armamentarium of nervous diseases.

In order that our study shall not be too far-reaching, I will limit myself to the consideration of the tendon reflexes only. The ones generally examined are the patellar, tendo Achillis, biceps, and triceps. The patellar and Achillis tendons will claim most of our attention.

*Mechanism.*—The theories regarding the mechanism of tendon reflexes have been various. The concurrence of the sensory and motor nerves in their production has long been recognized; the point of transfer has been the bone of contention. Descartes held that the shifting of the impulse from sensory to motor occurred in the pineal gland. The ganglia on the posterior spinal roots were, until very recently, regarded as the reflex center, but pathology has eliminated this theory from the probabilities. Much light was thrown upon the subject when, through the Hales-Whytt experiments, the spinal cord was found to be the true reflex center. Today the one theory accepted, and the one followed out by facts, is the following: Tapping the patellar tendon acts as a stimulus to the sensory nerve filaments in the tendon and muscle. This stimulus is carried centripetally by the afferent sensory nerves through the posterior spinal roots to the posterior horns of gray matter, thence conveyed through the posterior white columns to the anterior horns. In all probability, the transfer from sensory to motor conduction occurs in the large multipolar ganglion cells of the anterior horns.[1] The impulse is now carried centrifugally through the anterior spinal roots to the efferent motor nerve, and contraction of the quadriceps femoris muscle follows. This is called the reflex arc, and upon the integrity of this arc depends the consummation of the reflex. But this is not all: the brain has always been regarded as the great inhibitory center, and in the mechanism of reflex action the inhibition of the brain plays an important part. At the instigation of Weir Mitchell, Reichert of Philadelphia made experiments upon this point, which are here briefly summarized.

The spinal cords of twelve dogs were cut, either in the upper dorsal or lower cervical region, thus destroying all inhibitory fibers. The toes of one side, and the upper segment of the cord severed from the brain, were then subjected to the action of powerful stimuli, and yet, in all cases, without exception, there was no evidence that the knee-jerk was in any way affected by these excitants. Other experiments on the same point might be cited, but the conclusions arrived at are all the same; namely, that the exaggeration or reënforcement of the tendon reflexes is dependent upon some peculiar influence exerted by the cerebral centers. There are, probably, no special routes for these inhibitory fibers in passing to and from the brain; they follow the same paths as do the motor and sensory impulses. The point of transfer in the brain is, undoubtedly, the ganglion cells of the cortex corresponding to the motor center of the muscles in the anterior frontal convolution. Thus amended, the reflex arc of the patellar tendon would be: afferent sensory nerve to cord, through the posterior horn to the posterior white column. Here occurs a division, one impulse follows the sensory path to the brain, passes through the center of sensation to the motor center of the quadriceps femoris muscle, thence centrifugally through the internal capsule down the anterior and lateral pyramidal tracts to the efferent motor nerve. The

1. The substance of Rolando in the posterior horns is regarded by many as the point of transfer.

other division follows the course traced out a few moments ago. This represents, perhaps, the present status of our knowledge regarding the mechanism of tendon reflex action.

*Reflex Centers.*— The different tendon reflexes must each have a separate and distinct center in the cord as well as in the brain. By experimentation and traumatism to certain regions of the cord, whereby these centers have been destroyed, their exact localities have been determined. According to Westphal, Starr, Pick, and others, the spinal center governing the patellar tendon reflex is placed at the level of the second and third lumbar nerves. The center presiding over the tendo Achillis reflex (ankle clonus is placed at the level of the first to third sacral nerves, or lower part of the lumbar enlargement. According to Starr, the segment of the cord presiding over the triceps and biceps tendon reflexes is at the level of the sixth cervical nerve. The extensors of the hand are also located at the sixth cervical.

*Time.*— Waller, of England, has lately made an extended study on the physiological mechanism of tendon reflexes, and found that the time which elapsed in the production of the patellar reflex was 0.02–0.04, or $\frac{1}{50}$– $\frac{1}{25}$ of a second. It was immaterial whether he applied a stimulus to the patellar tendon or to the rectus femoris muscle, the time between the irritation and the contraction of the muscle, in both cases, remained the same. Gowers found the interval of time between the irritation and contraction to be $\frac{1}{30}$ of a second. Burckhardt found it to be 0.039, or $\frac{1}{25}$ of a second; Tschirjew, .032–.034, or about $\frac{1}{35}$ of a second; Brissaud, .04, or $\frac{1}{25}$ of a second; Eulenburg, .03 or $\frac{1}{33}$ of a second. The slight variation in the figures of the different observers must show that the experiments were most carefully carried out, and that the absolute interval of time for the production of the patellar tendon reflex to be not far from $\frac{1}{35}$ of a second.

*Nature of Tendon Reflex Action.*— Upon this point there exists diversity of opinion. Is the tendon reflex, so-called, a direct muscular contraction; secondly, a reflex, pure and simple, of the tendon; or, thirdly, a direct muscular contraction with the *conditio sine qua non* of the integrity of the reflex arc? Waller, basing his opinion on the experiments just mentioned, holds to the latter proposition. Erb champions the second,—that the contraction is a true reflex action, the stimulus being the excitation of the nerves in the tendon. Westphal held the third proposition to be the correct one, and believed that the contractions of the muscle do not necessarily depend on a simple reflex act from the tendons. The French school generally pin their faith in the reflex theory. Gowers, from the experiments made by Tschirjew, Burckhardt, and others, believes that the tendon reflexes are the product of reflex irritability, accompanied by local stimulation; in other words, that the phenomenon is due to muscle reflex irritability which has nothing to do with the tendons. This muscle irritability is nothing more than muscle tone, and is dependent on the spinal cord. Summing up, it would seem that the majority of observers uphold the direct muscular contraction theory dependent upon the functional integrity of the reflex arc. If this theory be the correct one, then the term "tendon reflex" is a misnomer, and we are obliged to look about for a better term. Gowers has already suggested tendon muscular phenomena, or myotatic contractions. The term tendon reflex has, however, become so rooted in our nomenclature, that nothing short of a revolution can efface it from our minds and text-books.

We are now in a position to judge of the importance of the tendon reflex as a diagnostic factor. Have we in it an agent which will give us positive evidence of disease or injury to the deeper nerve centers, or is it only of value in clinical histories, because of its universal adaptation? It may be said that, inasmuch as there exists difference of opinion as to its nature, there must exist discrepancies as to the condition of the several organs which produce it. This fear is groundless. In the majority of cases, in experienced hands, the examination of the tendon reflexes gives us information concerning afferent, efferent nerves, and cord, which would be difficult to obtain otherwise. Therefore, if there is virtue in this phenomenon, let us turn from the theoretical and problematical to the practical and actual, and determine the behavior of these reflexes in health and disease.

*Methods of Examination.*—In order that the reflex act shall give us a true index of the condition of the reflex arc, three things are necessary:

*First*—The mind of the patient must be neutral or passive,—at least must not be upon the proceedings which are taking place around him. We shall see, later on, that states or conditions of the mind influence the degree of reaction, and, hence, this factor must be eliminated while making our examination.

*Second*—The muscle and its tendon must be in a state of passive tension. It should not be flaccid nor taut. To obtain this desired condition, the limb should not be in an extreme flexed or extended position, but midway between extension and flexion.

*Third*—The force of the blow should be uniform, and occur at proper intervals. In examining the two sides, care must be taken to tap each tendon uniformly, if accurate results are desired.

Bearing these three points in mind, we will proceed to examine the patellar tendon.

The patient, unless otherwise reported, is to be seated in a chair, with his legs flexed at right angles upon the thigh. The leg of the side to be examined is crossed over the knee of the other leg, and the patient requested to let it hang as if dead. The operator then taps the patellar tendon, either with a percussion hammer, with the edge of his hand, or the tips of his fingers. There results contraction of the quadriceps femoris muscle, and the leg is jerked forwards. Care must be taken to tap the tendon about midway between its patellar and tibial insertions, and to accomplish this the leg should be bared, and the desired spot determined beforehand by palpation. I sometimes grasp the tendon lightly between the thumb and forefinger, so as to be sure that the blow falls on the right spot. Tapping the inner edge of the ligamentum patella will sometimes call forth a reaction when otherwise it fails to respond. This is the most universal method of examining the patellar tendon, but others are in vogue which have special indications for their employment. The operator may lift the leg from the floor, allowing it to hang over his arm, his hand resting upon the other knee of the patient. The tendon is then tapped. This method prevents the patient from exercising active tension over the muscles of the leg, and permits it to hang lifeless. The same ends are obtained if he sits on a table with the legs dangling from the side. Sometimes I have overcome this active tension by bringing the leg nearly to complete extension, and allowing the heel to rest on the floor. Tapping the tendon will cause the extension of the foot on the leg, the heel acting as a fulcrum. Another way is to grasp the foot with the hand, and raising the leg from the floor. By flexing the foot on the leg, a certain degree of tonus will be obtained, besides overcoming active tension. These two methods are especially applicable in aged and portly individuals, also when the patient is bedridden. Several methods are in practice which have for their object the distraction of the patient's mind while the tendons are being examined. With one leg crossed over the other, as in the first method, the patient is given examples to solve in mental arithmetic. While occupied with a problem, the tendon is tapped and the reaction noted. Another method, which has become famous, is Jendrassik's. The patient assumes the same position as in the foregoing, and is commanded to clinch his hands and pull them forcibly, while the physician taps the tendon. In cases where the reaction seemed absent altogether, or but slightly present, it can be elicited with some force. If no response is obtained, it can be assumed that the tendon reflex is abolished. If the patient is bedridden, the leg may be raised slightly at the knee, then percussed—or the hand may be applied to the rectus femoris, just above the patella, and examined. Erb has succeeded in obtaining a patellar clonus by forcibly pushing the patella downwards and holding it, thus making the rectus tense. The clonus will continue as long as tension is maintained. These are some of the more important ways of eliciting the phenomenon, designated knee-jerk by Gowers, knee phenomenon by Westphal, and patellar tendon reflex by Erb. The degree of reaction varies in health and disease. Generally speaking, if absent, or if reënforced or exaggerated, some disturbance or pathological condition is present.

*Ankle Clonus, Syn. Foot Clonus, Foot Phenomenon.*—The ankle clonus is a safer and more trustworthy guide than the patellar reflex, since in health it is totally absent, and only present when there is some organic lesion present in the cord, or some functional irritation to the cord or brain. It is generally obtained by grasping the foot with the right hand, the left hand supporting the lifted leg. By forcibly flexing the foot on the leg, thus making the gastrocnemius tense, the foot will be thrown into a series of clonic contractions, averaging from five to ten per second. These movements will continue as long as the foot is flexed on the leg, or as long as the calf muscles are under increased tension, and are nothing more than a series of Achillis tendon reflexes. Instead of provoking this clonus, the foot may be flexed on the leg, and the tendo Achillis percussed.

To examine the triceps tendon, the forearm is held slightly flexed on the upper arm, and the tendon slightly percussed just above the olecranon process. It is sometimes very difficult to elicit any response, but in disease, with care and a percussion hammer, a sudden extension of the forearm can be obtained. To examine the biceps, hold the arm slightly flexed as before, and seek the biceps tendon just above the elbow joint ; percuss slightly, and a sharp flexion of the forearm will follow. For the superficial and deep flexors of the fingers, strike the tendons just centrad of the wrist joint. These arm reflexes are generally absent in health, and only present when there exist organic lesions in the central nervous system. Another muscular reflex sometimes sought for is the masseter. It is obtained by placing a lead pencil in the mouth, resting it upon the molar teeth. Strike the pencil near its emergence from the mouth, and a sharp quick upward movement of the inferior maxilla will take place. This reflex is only present, according to Charcot, in bulbar paralysis.

In all these cases, especially where the reflex is very weak or absent, we must be very careful not to mistake the movement of the limb, caused by the shock of the blow, for the reaction.

In order to study the clinical significance of the different reflexes in disease, we must know beforehand how they act in health, or when there is no lesion, organic or functional, of the various systems of the body which beget them.

In health, the patellar reflex is fickle, inconstant, capricious, dependent in great measure upon the whim or mental condition of the individual. No absolute rule can be laid down, since in no two persons does it act uniformly. If the necessary precautions are heeded, the reaction should be a determined resolute forward movement of the leg, describing, according to Ziehen, an arc of a circle of eleven to forty degrees, or, better, fifteen to thirty degrees. The behavior may be said to be "good-natured." It is, as a rule, always present in health, although exceptional cases are sometimes met with. Pelizaeus examined 2,403 school children, and found it present in every one. Jendrassik examined 1,000 persons, chiefly adults, and found it absent in only one case, and that was a case of diabetes, an affection in which the reflex is generally absent. Zenner, of Cincinnati, examined 1,672 individuals, and found it absent in five, two of whom were affected with locomotor ataxia, so that really in 1,670 healthy individuals it was absent in only three. It is sometimes very difficult to elicit this phenomenon in the very young and very old, and much care must be taken not to mistake the shock of the blow for a weakened tendon reflex. Lombard made some interesting studies on the patellar reflex in health, and came to the following conclusions: It varies with the time of day, being highest in the morning; is increased after each meal; is diminished by muscular or mental fatigue; is increased by mental activity; is influenced by the state of the weather, being increased by a fall of temperature and decreased by a rise; the emotions, as joy, expectation, in short, whatever buoys up the spirits, increases, while sadness, sorrow, anger, hatred, or whatever depresses the mind, diminishes the activity of the reaction. Jendrassik's discovery, that the clinching of the hands while the reflexes were being examined, would increase it, led Mitchell and Lewis to make further investigations. They found that any decided movement on the part of the individual, or any sensation, if perceived simultaneously with the tapping of the tendon, would exaggerate the reflex. Also, that if a galvanic current were passed through the frontal lobes of the brain, the reflexes would be increased. Bowditch and Warren took up the thread of these experiments, and showed that the interval of time elapsing between the volitional movement of the individual and the tapping of the tendon became a factor in influencing the reflex. If that interval was 0.4 of a second or less, the reflex would be increased; if 1.7 seconds or more, the movement would exert no influence whatever.

Now, what does all this mean? Does it destroy all hope of employing this phenomenon as a symptom of disease, because in health it varies as the play of colors in a brilliant, or must we regard some of these variances as pathological, and the reflex as abnormal?

From our own studies we have found it almost universally present in health, or when the reflex arc is intact, hence it is but fair to conclude that its absence forebodes some disturbance in the course of this arc, and that disturbance means—disease. On the other hand it was found to vary in intensity, but when greatly exaggerated or reënforced, there was usually present some functional disturbance or heightened activity of the higher nerve centers. But allowing all possible chance for error, we will grant that an increased patellar reflex, if not too extreme, cannot *per se* be regarded as indicative of disease.

The ankle clonus gives us no chance whatever to doubt of its value as a diagnostic symptom. It is either present or absent, presents no variations, is independent of the whim or fancy, and signifies at all hours and under all circumstances, that a neuropathic lesion is present somewhere in the animal economy. It is pathognomonic of disease of the lateral pyramidal tracts, either occurring primarily or secondarily by a descending degeneration of these tracts as a result of some cerebral insult. It will not be found, though, when there exists at the same time some disturbance in its reflex arc. An increased patellar reflex, if coupled with ankle clonus, becomes, then, an important diagnostic factor, and signifies some obstruction or irritation along the highway of nervous conduction.

The biceps and triceps tendon reflexes, likewise the flexors of the fingers and masseter, occur so rarely in health, that their presence augur disease.

To recapitulate, the normal patellar tendon reflex occupies middle ground, or may be designated good-natured; the ankle clonus, biceps and triceps tendon reflexes, are always absent.

Having observed these reflexes in health, we can now judge of their behavior in disease. The patellar reflex occupying a mean position in health, must swerve to the extremes, either markedly exaggerated, or abolished. Let us study, first, the exaggerated reflex. If the patellar tendon be but slightly percussed, the leg will fly up instantly in a snappy, threatening manner, defining an arc of a circle of thirty-five to seventy-five degrees. If the taps on the tendon follow each other in quick succession, the leg will be thrown into clonic contractions, gradually diminishing, until the leg is held in tonus. With a little experience, the difference between a normal and an abnormal reflex can be easily determined.

We have found that an exaggerated or snappy reflex occurs when there is loss of inhibitory action, and, secondly, when the nerve centers are in a state of over-stimulation or excitation.

Loss of inhibition results when there is either complete or partial obstruction of nerve impulses, coming or going from the spinal reflex center to the brain, or *vice versa*. It is consequent to organic disease of the cord, seated in or affecting in any way the anterior or lateral pyramidal tracts, and, secondly, to injuries of the cord cephalad of the reflex centers.

Over-stimulation and excitation of the nerve centers occur in functional neuroses and psychoses.

We find loss of inhibition in myelitis, where the white substance of the cord has undergone sclerotic change; in amyotrophic lateral sclerosis and spastic paraplegia, where a similar change has taken place in the pyramidal tracts; and in multiple sclerosis, where sclerotic patches are distributed throughout the white substance of the cord. The reflexes are likewise exaggerated in syringo- and hydro-myelia, where, on account of the distension of the intra-spinal cavities, pressure is exerted on the neighboring pyramidal tracts, and in many cases results in the new formation of connective tissue: In hematomyelia and hematorrhacis (or spinal apoplexy), pachymeningitis hemorrhagica interna, and spinal tumors, owing to the pressure exerted on the cord by the escaping blood or new formation of tissue. In these affections, where pressure is the disturbing agent, the insult must be cephalad of the reflex centers, and not destroy the reflex arc. In pachymeningitis hypertrophica cervicalis, where we have an undue thickening and hypertrophy of the membranes, the cord is in great measure destroyed, and a descending degeneration of the pyramidal tracts follow.

In Brown-Sèquard's spinal paralysis we find the reflexes exaggerated on the side of the motor paralysis, because on this half of the cord there is loss of continuity, and, hence, the inhibitory fibers must necessarily be severed.

In arthritic muscular atrophies, where the wasting is due to some alteration in the nutrition of the ganglion cells of the cord, we find increased reflexes only in the limb whose joints have suffered injury.

We also find the reflexes reënforced in certain cerebral affections, as in cerebral apoplexy, cerebral embolism, and acute encephalitis. As a result, we have hemiplegia, with secondary degeneration of the pyramidal tracts. The increased reflexes pertain to that side corresponding with the motor paralysis. In hematomas of the dura mater, if seated over the motor centers in the brain, the reflexes on the opposite side of the body will be exaggerated. In chronic hydrocephalus and senile dementia the exaggeration is due to the long continued irritation and the slight organic changes which, of necessity, follow.

In all these cases of organic disease of the cord and brain, we generally find ankle clonus along with the exaggerated patellar tendon reflex.

Among the functional disturbances or neuroses we often meet the heightened patellar reflex, but less often the ankle clonus. Absolute reliance cannot be placed upon the reflex phenomena in these cases, although many times their behavior will aid us in distinguishing between organic and functional disease. In hysteria we find that the patellar reflex is greatly exaggerated, especially in those individuals offering same paralysis, contracture, atrophy, or after convulsions.

In epilepsy the same condition is observed in sixty per cent. of all cases, especially after an attack. During the intervals the reflexes approach their normal activity. Oliver found that the knee-jerk is sometimes present, and sometimes absent, but the ankle clonus is always present. Beevor found that after seventy attacks the knee-jerk increased and clonus present thirty-eight times, knee-jerk diminished and clonus absent, twenty-four times.

In neurasthenia, paramyoclonus, and tetanus, the exaggerated reflexes become an important diagnostic factor, especially in the two latter affections, where they are present to an excessive degree. Simply touching the tendons will throw the whole body into clonic spasms.

In the various psychoses, as paranoia, mania, and the affective forms, we meet the exaggerated reflexes, portraying somewhat the state of the cerebral centers.

Lastly, in some acute infectious processes, as in cholera, typhoid fever, dysentery, etc., the reflexes will be found at times to be increased.

*Abolition of the Reflexes.*—From our study of the mechanism of reflex action, we learnt that the reflexes are abolished whenever the reflex arc is impaired or broken ; in other words, when there is present some organic lesion in the afferent or efferent nerves or cord, particularly in the posterior white columns and multipolar ganglion cells of the anterior horns. No response is obtainable in these cases, even with Jendrassik's method, and the leg hangs limp and lifeless, the muscles inert, possessing no myotatic irritability. The reflex may be termed disgusted, and the arc may be likened to a broken circuit.

We find this state of affairs in the simple neuritic processes, in those forms due to toxic agents, as alcohol and arsenic, in those of endemic origin, as beri-beri, and in those forms of neuritis occurring concomitant with infective processes, as in diphtheritic paralysis. In all these varieties the reflex arc is destroyed by virtue of the non-conductibility of the afferent and efferent nerves.

Among the diseases of the spinal cord we find the reflexes most generally absent in locomotor ataxia. Here there is a sclerotic tissue development in the posterior white columns, and the arc is consequently destroyed. In poliomyelitis anterior, and progressive muscular atrophies of spinal origin, as the Duchenne-Aran and Charcot-Tooth types, the reflex arc is likewise tampered with on account of the destruction of the multipolar ganglion cells of the anterior horns. In Friedreich's disease, or hereditary ataxia, we meet obstruction, owing to sclerotic changes in the posterior and lateral white columns. In chorea molle or paralytic chorea, chronic ergotism and diabetes, the reflexes are unaccountably absent. In trauma or injury to the cord at the seat of the reflex, whereby the centers are destroyed, the reflex will, of course, be absent. In all these cases abolition of the reflexes is due to some obstacle in the path of the reflex arc.

There are also some diseases in which the reflexes vary, in some stages present and in some absent. This is true of spinal and cerebral meningitis. In the first stage, stage of irritation, the reflexes are increased, while in the second, stage of paralysis, they are absent.

In progressive paralysis some observers have found the reflexes abolished, and some have found them increased. Ziehen found them absent in 22 per cent., exaggerated in 32 per cent. Zenner found them absent in nearly every case examined.

In idiocy the reflexes also vary. Wildermuth found exaggeration in 16 per cent., abolition in 23.5 per cent. Ziehen found them reënforced in over 50.

Exaggeration of Tendon Reflexes.

- Organic Disease.
  - Spinal Cord.
    1. Myelitis.
    2. Amyotrophic lateral sclerosis.
    3. Paraplegia spastica.
    4. Multiple sclerosis.
    5. Syringomyelia and hydromyelia.
    6. Hematomyelia and hematorrhacis.
    7. Spinal tumors.
    8. Pachymeningitis hemorrhagica interna.
    9. Pachymeningitis cervicalis hypertrophica.
    10. Brown-Séquard's spinal paralysis
    11. Arthritic muscular atrophies.
  - Brain
    1. Hemiplegia
       - Cerebral apoplexy.
       - Cerebral embolism, thrombosis.
       - Acute encephalitis.
    2. Hematoma.
    3. Hydrocephalus.
    4. Senile dementia.
- Functional Disease.
  1. Hysteria.
  2. Epilepsy.
  3. Neurasthenia.
  4. Paramyoclonus.
  5. Tetanus.
  6. Psychoses.
  7. Infectious processes.

Abolition of Tendon Reflexes.

1. Neuritis.
   - Simple.
   - Toxic.
   - Endemic.
   - Infective.
2. Locomotor ataxia.
3. Poliomyelitis anterior.
4. Spinal muscular atrophies.
5. Hereditary ataxia (Friedreich).
6. Chorea molle.
7. Chronic ergotism.
8. Diabetes mellitus.
9. Traumatism.

Abolition and Exaggeration.

1. Meningitis
   - Spinal.
   - Cerebral.
2. Dementia paralytica.
3. Idiocy.

*Bibliography.*—C. F. Hodge, *Am. Jour. Psychology*, 1890, No. 2; E. T. Reichert, *Jour. of Nerv. and Ment. Dis.*, 1890, No. 2; Jendrassik, *Deutsche Archiv. f. Klin. Med.*, 1883, Band 33, page 177; Mitchell and Lewis, *Philadelphia Med. News*, Feb., 1886; Mitchell, *Philadelphia Med. News*, June, 1888; Lombard, *Am. Jour. Psychology*, Oct., 1887; Bowditch and Warren, *Boston Med.-Surg. Jour.* 1888; Pick, *Archiv. f. Psychiatrie*, 1889, No. 3; Waller, *Jour. of Physiology*, 1890, Vol. XI., No. 485; Ziehen, *Correspondenz Blätter des Allg. Ärztl. Verein von Thüringen*, 1889, No. 1; Charcot, Vol. IX. *Oevures Complètes*; Gowers, *Diseases of Nervous System*; Seeligmüller, *Krankheiten des Nerven System*; Zenner, *Lancet-Clinic*, 1889, Nov. 16; Bastian, Quain *Dictionary of Medicine*; Strümpell, *Krankheiten des Nerven System.*

382 Virginia Street.

